# Job Loss, Unemployment and the Incidence of Hazardous Drinking during the Late 2000s Recession in Europe among Adults Aged 50–64 Years

**DOI:** 10.1371/journal.pone.0140017

**Published:** 2015-10-07

**Authors:** Marina Bosque-Prous, Albert Espelt, Luis Sordo, Anna M. Guitart, M. Teresa Brugal, Maria J. Bravo

**Affiliations:** 1 Agència de Salut Pública de Barcelona, Barcelona, Spain; 2 Institut d'Investigació Biomèdica Sant Pau (IIB Sant Pau), Barcelona, Spain; 3 Universitat Pompeu Fabra (UPF), Barcelona, Spain; 4 CIBER de Epidemiología y Salud Pública (CIBERESP), Madrid, Spain; 5 Departament de Psicobiologia i Metodologia en Ciències de la Salut, Facultat de Psicologia, Universitat Autònoma de Barcelona, Bellaterra (Cerdanyola del Vallès), Spain; 6 Departamento de Medicina Preventiva y Salud Pública. Facultad de Medicina. Universidad Complutense de Madrid, Madrid, Spain; 7 Centro Nacional de Epidemiología. Instituto de Salud Carlos III. Madrid, Spain; National Institute of Health, ITALY

## Abstract

**Background:**

To estimate the incidence of hazardous drinking in middle-aged people during an economic recession and ascertain whether individual job loss and contextual changes in unemployment influence the incidence rate in that period.

**Methods:**

Longitudinal study based on two waves of the SHARE project (Survey of Health, Ageing and Retirement in Europe). Individuals aged 50–64 years from 11 European countries, who were not hazardous drinkers at baseline (n = 7,615), were selected for this study. We estimated the cumulative incidence of hazardous drinking (≥40g and ≥20g of pure alcohol on average in men and women, respectively) between 2006 and 2012. Furthermore, in the statistical analysis, multilevel Poisson regression models with robust variance were fitted and obtained Risk Ratios (RR) and their 95% Confidence Intervals (95%CI).

**Results:**

Over a 6-year period, 505 subjects became hazardous drinkers, with cumulative incidence of 6.6 per 100 persons between 2006 and 2012 (95%CI:6.1–7.2). Age [RR = 1.02 (95%CI:1.00–1.04)] and becoming unemployed [RR = 1.55 (95%CI:1.08–2.23)] were independently associated with higher risk of becoming a hazardous drinker. Conversely, having poorer self-perceived health was associated with lower risk of becoming a hazardous drinker [RR = 0.75 (95%CI:0.60–0.95)]. At country-level, an increase in the unemployment rate during the study period [RR = 1.32 (95%CI:1.17–1.50)] and greater increases in the household disposable income [RR = 0.97 (95%CI:0.95–0.99)] were associated with risk of becoming a hazardous drinker.

**Conclusions:**

Job loss among middle-aged individuals during the economic recession was positively associated with becoming a hazardous drinker. Changes in country-level variables were also related to this drinking pattern.

## Introduction

The late 2000s economic crisis has produced important socioeconomic changes in most European countries, such as increasing unemployment rates and a drop in gross domestic product [1]. Over the last decades, social consequences of economic crises have been associated with increase in health problems, including alcohol-related morbidity and mortality [2,3]. Although the World Health Organization has expressed concerns about the impact of the current economic crisis on alcohol death rates [4], the evidence on the consequences of this recession on alcohol use is still limited.

Risky drinking patterns influence the burden of disease and are related to unintentional and intentional injury, violence, stroke and sudden cardiac death [5,6]. Although overall alcohol consumption decreases during times of economic recession, risky patterns can rise [7–12]. There seems to be a relationship between the individual changes in the financial situation of individuals during economic downturns (e.g. job loss, reduced income), and alcohol abuse and dependence [13,14]. Nevertheless, evidence is not conclusive. While some studies find a positive relationship [15–18], others find an inverse one or no association [19,20]. The discrepancies on the consequences of economic crisis and unemployment could be partly explained by country differences in social protection and political and social measures implemented by governments [1,21,22]. Socioeconomic variables at country level may have an effect on alcohol consumption that is not captured by the variables at individual level [14]. Therefore, studies using multilevel regression models are needed to take into account both individual and contextual variables.

Several mechanisms are involved in the relationship between economic downturns and alcohol consumption. Alcohol consumption in a country could decrease during economic crisis because of a global loss of purchasing power. However, certain sub-populations could increase their consumption due to stress [17,23,24]. General life stressors such as job loss/change or problems at work are related to alcohol consumption and increase the risk for alcohol use disorders [24]. Likewise self-reported job strain is related with increased alcohol intake [25].

Although some age-groups might be more vulnerable than others to the harmful effects of drinking, most studies aiming at analyzing the influence of job loss on alcohol consumption have focused on active population of all ages. Alcohol consumption generally declines with age but older drinkers typically consume alcohol more frequently than other age groups [26]. Middle-aged and older individuals have greater risk of alcohol-related problems than younger age groups, as they are more vulnerable to the adverse effects of alcohol due to age-related changes [26,27], such as increased sensitivity and decreased tolerance to alcohol and a slower metabolism [28,29]. Economic downturns and shrinking job markets have a substantial negative impact on the re-employment opportunities among individuals approaching retirement [30,31]. This adverse context could place them at higher risk of adopting dangerous drinking patterns. Studies on the factors influencing heavy alcohol intake in middle-aged people have been recommended [27,32].

The late recession in Europe, and the changes it has brought in the working status of people, is likely to be affecting the patterns of alcohol consumption, in particular in the population aged 50–65 years. Consequently, our aims were: 1) to estimate incidence of hazardous drinking in middle-aged people during a period of economic recession; and 2) to ascertain whether individual job loss and contextual changes in employment status during this period influence the incidence of hazardous drinking among this population.

## Methods

### Study Design and Sample

We used a longitudinal design based on data from waves 2 and 4 (2006–07 and 2011–12, hereinafter called 2006 and 2012) of the Survey of Health, Ageing and Retirement in Europe (SHARE) project [33,34], for 11 European countries that participated in both waves: Austria, Belgium, Czech Republic, Denmark, France, Germany, Italy, Netherlands, Spain, Sweden and Switzerland. Each wave of the SHARE survey consisted on a face-to-face computer-assisted interview (CAPI), supplemented by a self-administered questionnaire. The survey of each wave covered economic, social and health factors that accompany and influence ageing processes (for further details on the survey methodology, see www.share-project.org [35–37]). Data from waves 1 and 3 were not included in the study as the alcohol questions in those two waves were different and not comparable. Poland participated in the SHARE project, but its data were excluded from the study to avoid that its extreme values on both prevalence of hazardous drinking and contextual variables could drive the results. Sampling was performed independently in each country; all countries obtained a probabilistic sample, although the exact sample design differed slightly between countries. The inclusion criteria for this study were being 50- to 64-years old and not being a hazardous drinker at baseline (2006–2007). People with no data on alcohol consumption at baseline were excluded (1.5%). The final sample size was of 7,615 individuals.

### Dependent variable

The dependent variable was the incidence of hazardous drinking. To calculate individual daily alcohol consumption, we combined individual’s responses to questions on drinking frequency during the previous 3 months (“How often did you drink any alcoholic beverages, like beer, cider, wine, spirits or cocktails?”) and typical quantity per occasion (“On the days you drank during the past three months, about how many drinks do you have?”). Hazardous drinking was defined as an average daily consumption of ≥40g and ≥20g of pure alcohol during the previous 3 months, in men and women, respectively [38,39]. The incidence of hazardous drinking was calculated using answers given by participants during visits at baseline (2006–07) and follow-up (2011–12). Participants who said, at baseline, that they had not consumed alcohol during the three months prior to the interview were classified as abstainers, and the ones who drank but were not hazardous drinkers were classified as low-risk drinkers.

### Independent variables

The individual-level independent variables were: sex, age, country of residence, employment status [employed; unemployed; other (retired, homemaker, sick or disabled)], changes in employment status (employed at baseline and follow-up; employed at baseline, unemployed at follow-up; unemployed at baseline, employed at follow-up; other status), educational level (lower secondary education or less; upper secondary education or more) and self-perceived health (good, very good or excellent; fair or poor).

The country-level independent variables were: a) changes in unemployment rate between 2006 and 2010 (dichotomous: decrease, increase) [40]; b) percentage of increase in Gross Domestic Product (GDP) between 2006 and 2010 (continuous) [41]; c) percentage of increase in social protection expenditure as a percentage of GDP between 2006 and 2010 (continuous) [41]; d) percentage of increase in household disposable income between 2006 and 2010 (continuous) [40]; e) standardized Gini index for 2007 (continuous) [41]; f) changes in Gender Inequality Index between 2005 and 2010 (dichotomous: decrease, increase) [42]; g) standardized degree of alcohol advertising restriction, 2008 (continuous) (S1 Table) [43,44]; h) other standardized alcohol control policies, 2006 (continuous) (S2 Table) [45]; i) drinking patterns score, 2005 (dichotomous: low-risk drinking patterns, risk drinking patterns) [43]. For country-level variables, we used data from 2006 and 2010 where available for all participating countries. However, where no information was available for either year, we used data from the nearest year.

### Ethics Statement

During waves 1 to 4, SHARE has been repeatedly reviewed and approved by the Ethics Committee of the University of Mannheim. In addition wave 4 was reviewed and approved by the Ethics Committee of the Max Planck Society in 2012. All information in SHARE is pseudo-anonymised and therefore the identification of individual persons is not possible. All respondents have been informed about the storage and use of the data and about their right to withdraw their consent. Written consent was given by the respondents for their information to be stored in the database and used for research when required by national or regional data protection laws.

### Statistical analysis

The sample distribution at the beginning of the follow-up period was calculated for each individual variable. Follow-up bias was assessed among the 7,615 individuals at baseline. The cumulative incidence of hazardous drinking between 2006 and 2012 was estimated for each individual-level independent variable. To test for association between the incidence of hazardous drinking and changes in employment status, while accounting for individual- and country-level variables, we fitted multilevel Poisson regression models with robust variance, which yielded risk ratios (RR) and 95% Confidence Intervals (95%CI). All analyses were adjusted for length of follow-up (i.e. time between the two interviews), which was measured in years. First, we estimated the variability of the incidence of hazardous drinking between countries (empty model: Model 0). Second, we fitted several bivariate Poisson regression models with robust variance, including in each model the dependent variable (incidence of hazardous drinking) and one independent variable (Model 1). The final adjusted model (Model 2) was fitted by including all individual- and country-level variables and eliminating, one at a time, each of the contextual variables that were not statistically significant. Thus, the final adjusted model included all individual variables, as well as the country-level variables that were statistically significant in the multivariate analysis. Statistical analyses were conducted using STATA 13.0 and HLM6.

## Results

Table 1 shows the baseline characteristics of the study sample. At baseline, the mean age of the study cohort was 57.6 years. Around 36% of individuals interviewed at wave 2 were excluded from the study for various reasons: a) they had no follow-up interview because of dying between waves or moving to an unknown address/refused to continue participating in the project); and b) they lacked data in wave 4 concerning any of the main variables. In all cases, they were considered lost to follow-up. The percentage of individuals lost to follow-up varied among countries from 24.9% in Switzerland to 50.9% in Czech Republic. Of the 7,615 subjects that were followed up from wave 2 to wave 4, 56% were women, 61.9% had completed upper secondary education or more, 75.1% reported that their self-perceived health was good, very good or excellent, 53.2% had a job and 4.8% were unemployed. With regard to alcohol consumption, 75.3% of individuals were low-risk drinkers and 24.7% were abstainers at baseline.

**Table 1:** Distribution of participants according to independent variables at baseline and follow-up. Survey of Health, Ageing and Retirement in Europe project (SHARE), 2006–2012.

	Follow-up		Lost to follow-up			
	n	%	n	%	p-value	% of individuals lost to follow-up
***Age***						
50–54 years	2,044	26.9	1,345	30.7	<0.001	39.7
55–59 years	2,766	36.3	1,604	36.7		36.7
60–64 years	2,805	36.8	1,427	32.6		33.7
***Sex***						
Women	4,264	56.0	2,323	53.1	0.002	35.3
Men	3,351	44.0	2,053	46.9		38.0
***Country***						
Austria	270	3.5	239	5.4	<0.001	47.0
Belgium	911	12.0	415	9.5		31.3
Czech Republic	708	9.3	735	16.8		50.9
Denmark	846	11.1	375	8.6		30.7
France	843	11.1	432	9.9		33.9
Germany	668	8.8	474	10.8		41.5
Italy	852	11.2	337	7.7		28.3
Netherlands	746	9.8	412	9.4		35.6
Spain	533	7.0	303	6.9		36.2
Sweden	727	9.5	485	11.1		40.0
Switzerland	511	6.7	169	3.9		24.9
***Employment status***						
Employed	4,052	53.2	2,377	54.3	0.093	37.0
Unemployed	368	4.8	220	5.0		37.4
Retired	1,761	23.1	995	22.7		36.1
Homemaker	930	12.2	467	10.7		33.4
Sick or disabled	415	5.5	270	6.2		39.4
Other	89	1.2	47	1.1		34.6
***Educational level***						
Lower secondary education or less	2,902	38.1	1,755	40.1	0.031	37.7
Upper secondary education or more	4,713	61.9	2,621	59.9		35.7
***Self-perceived health***						
Good, very good or excellent	5,721	75.1	3,165	72.3	0.001	35.6
Fair or poor	1,894	24.9	1,211	27.7		39.0
						
*Total*	*7*,*615*	*100*.*0*	*4*,*376*	*100*.*0*		36.5

p-value indicates whether the percentage of individuals in each category of the variables differ when comparing the individuals followed up versus the ones not followed.

During follow-up, 505 subjects became hazardous drinkers. The cumulative incidence of hazardous drinking during the 6 years of follow-up (2006–2012) was of 6.6 per 100 persons (95%CI = 6.1–7.2), with some differences between countries. The incidence varied from 5.1% in Germany or 5.2% in Austria and Denmark to 7.7% in Italy and Spain or 8.2% in Belgium, although the differences among countries were not significant (Table 2). The cumulative incidence of hazardous drinking during the follow-up in people who were employed at baseline and follow-up was of 6.4 per 100 persons (95%CI = 5.5–7.4), whereas in people who were employed at baseline but unemployed at follow-up it was of 10.1 per 100 persons (95%CI = 6.0–16.3).

**Table 2:** Cumulative incidence of hazardous drinking in 11 European countries participating in the Survey of Health, Ageing and Retirement in Europe project (SHARE), 2006–2012.

			cumulative incidence (2006–12)	
	N	cases	%	[95% CI]
***Country***				
Austria	270	14	5.2	[3.1–8.6]
Belgium	911	75	8.2	[6.6–10.2]
Czech Republic	708	53	7.5	[5.8–9.7]
Denmark	846	44	5.2	[3.9–6.9]
France	843	51	6.0	[4.6–7.9]
Germany	668	34	5.1	[3.7–7.0]
Italy	852	66	7.7	[6.1–9.7]
Netherlands	746	52	7.0	[5.3–9.0]
Spain	533	41	7.7	[5.7–10.3]
Sweden	727	47	6.5	[4.9–8.5]
Switzerland	511	28	5.5	[3.8–7.8]
***Age groups***				
50–54 years	2,044	120	5.9	[4.9–7.0]
55–59 years	2,766	191	6.9	[6.0–7.9]
60–64 years	2,805	194	6.9	[6.0–7.9]
***Sex***				
Women	3,993	271	6.4	[5.7–7.1]
Men	3,117	234	7.0	[6.2–7.9]
***Employment status***				
Employed	3,764	288	7.1	[6.4–7.9]
Unemployed	341	27	7.3	[5.1–10.5]
Retired	1,640	121	6.9	[5.8–8.2]
Homemaker	887	43	4.6	[3.4–6.2]
Sick or disabled	396	19	4.6	[2.9–7.1]
Other	82	7	7.9	[3.8–15.6]
**Changes in employment status**				
Employed at baseline–Employed at follow-up	2,429	166	6.4	[5.5–7.4]
Employed at baseline–Unemployed at follow-up	125	14	10.1	[6.0–16.3]
Unemployed at baseline–Employed at follow-up	61	5	7.6	[3.2–17.0]
Other status	4,494	320	6.6	[6.0–7.4]
***Educational level***				
Lower secondary education or less	2,712	190	6.5	[5.7–7.5]
Upper secondary education or more	4,398	315	6.7	[6.0–7.4]
***Self-perceived health***				
Good, very good or excellent	5,317	404	7.1	[6.4–7.8]
Fair or poor	1,793	101	5.3	[4.4–6.4]
*Total*	7,615	505	6.6	[6.1–7.2]

As shown in Table 3, the variability of the cumulative incidence of hazardous drinking between countries (Model 0) was low (variance = 0.01; p-value = 0.157). Among variables analysed at individual level, an association was found in the bivariate analysis (Model 1) between the incidence of hazardous drinking: reporting fair or poor self-perceived health [RR = 0.75 (95%CI = 0.56–0.99)] and changing the employment status from being employed at baseline to unemployed at follow-up [RR = 1.56 (95%CI = 1.08–2.25)]. At country level, the incidence of hazardous drinking was associated with an increase in the unemployment rate [RR = 1.34 (95%IC = 1.19–1.50)], a greater percentage of increase in the Household Disposable Income [RR = 0.97 (95%CI = 0.96–0.99)] and an increase or no changes in the Gender Inequality Index [RR = 0.76 (95%CI = 0.68–0.85)].

**Table 3:** Risk ratios of incidence of hazardous drinking using multilevel Poisson regression models with robust variance. SHARE project, 2006–2012.

	Model 0 (empty)	Model 1 (bivariate)		Model 2 (multivariate)	
		RR	[95%CI]	RR	[95%CI]
***Individual variables***					
**Age**		1.01	[0.99–1.04]	**1.02**	**[1.00–1.04]**
**Sex**					
Women		1		1	
Men		1.10	[0.78–1.54]	1.07	[0.76–1.49]
**Educational level**					
Lower secondary education or less		1		1	
Upper secondary education or more		1.07	[0.92–1.24]	1.09	[0.92–1.30]
**Self-perceived health**					
Excellent, very good or good		1		1	
Fair or poor		**0.75**	**[0.56–0.99]**	**0.75**	**[0.60–0.95]**
**Changes in employment status**					
Employed at baseline–Employed at follow-up		1		1	
Employed at baseline–Unemployed at follow-up		**1.56**	**[1.08–2.25]**	**1.55**	**[1.08–2.23]**
Unemployed at baseline–Employed at follow-up		1.19	[0.45–3.13]	1.25	[0.47–3.30]
Other status		1.02	[0.76–1.36]	0.98	[0.76–1.28]
**Follow-up time**		0.89	[0.57–1.40]		
***Country-level variables***					
**Changes in unemployment rate**					
Decrease in Unemployment rate		1		1	
Increase in Unemployment rate		**1.34**	**[1.19–1.50]**	**1.32**	**[1.17–1.50]**
**Percentage of increase in Gross Domestic Product (GDP)**		1.00	[0.98–1.01]		
**Percentage of increase in Expenditure on Social Protection as % GDP**		1.01	[1.00–1.02]		
**Percentage of increase in Household Disposable Income**		**0.97**	**[0.96–0.99]**	**0.97**	**[0.95–0.99]**
**GINI index**		1.03	[0.92–1.14]		
**Changes in Gender Inequality Index**					
Decrease in Gender Inequality Index		1			
Increase or no changes in Gender Inequality Index		**0.76**	**[0.68–0.85]**		
**Degree of alcohol advertising restrictions**		0.94	[0.86–1.02]		
**Other alcohol control policies**		0.99	[0.93–1.06]		
**Patterns of Drinking Score**		0.95	[0.75–1.20]		
Variance	0.010	(p-value = 0.157)		0.001	(p-value >0.500)

After adjusting for individual and country-level variables (Model 2; Table 3), we observed an association between losing one’s job during the follow-up period and becoming a hazardous drinker [RR = 1.55 (95%CI = 1.08–2.23)]. Participants’ age also tended to be positively associated with the incidence of hazardous drinking, with older individuals being more likely to become hazardous drinkers [RR = 1.02 (95%CI = 1.00–1.04)]. We also observed an inverse association between self-perceived health status and the incidence of hazardous drinking: individuals with fair or poor self-perceived health were less likely to become hazardous drinkers [RR = 0.75 (95%CI = 0.60–0.95)].

While the incidence of hazardous drinking did not vary markedly between countries, it was higher in countries where the unemployment rate increased between 2006 and 2010 [RR = 1.32 (95%CI = 1.17–1.50)], and lower in countries where household disposable income increased more between 2006 and 2010 [RR = 0.97 (95%CI = 0.95–0.99)].

## Discussion

Accumulated incidence of hazardous drinking among middle-aged people was 6.6 per 100 persons from 2006 to 2012 (1.1 cases per 100 persons each year), with some differences between countries that were not statistically significant. Our study observed that job loss in people aged 50 to 64 years during a period of economic recession is associated with increased alcohol consumption. Moreover, increases in the national unemployment rate are also associated with higher incidence of hazardous drinking (32% more). A one percentage-point increase in household disposable income is associated with a 3% decrease in the incidence of hazardous drinking. As far as we know, this is the first study analysing the effects of the late 2000s economic crisis on alcohol consumption in several European countries that focuses on middle-aged people, who present vulnerabilities to both alcohol intake and re-employment.

### Strengths and Limitations

Both, individual (e.g. changes in participants´ employment status) and country level factors (e.g. unemployment rate) may be affected by an economic crisis and both should be taken into account to better analyze changes in alcohol consumption during an economic downturn. The main strength of our study is the consideration of the two types of factors in the analysis, which is not usual in this research field [46]. Our study presents some limitations. Firstly, we could not determine whether changes in employment status lead to hazardous drinking or *vice versa*, since there were no data on the date of changes in employment status, and data on drinking only capture the three months prior to the follow-up interview. Secondly, 36.5% of individuals did not have a follow-up interview and were excluded from the study, which indicates a potential differential follow-up bias. In the follow-up bias assessment, we found some differences between those who were lost to follow-up and those who were not for some individual-level variables, but not for employment status (Table 1). We analysed each cross-sectional survey separately (incl. all participants, regardless of follow-up status), and observed no statistically significant difference between baseline and follow-up in the prevalence of hazardous drinking in these variables. Moreover, our conclusions are further supported by the fact that the risk of hazardous drinking among unemployed compared to employed participants was 1.42 (95%CI: 1.13–1.80) in the entire 2011–12 sample, and just 1.19 (95%CI: 0.88–1.60) in the 2006–07 sample. Thirdly, the use of individual self-reported data might have led to underreporting bias in some population groups, such as unemployed hazardous drinkers who wish to avoid a stigma that would hinder job searching [19]. Fourthly, the incidence of hazardous drinking could be underestimated because of survival bias: individuals with a history of hazardous drinking but who gave up drinking following development of a serious alcohol-related illness are more likely to die during follow-up than those without serious illnesses. Fifthly, while we controlled for various individual-level factors related to alcohol consumption, we were not able to account for participants’ previous consumption of alcohol or other substances due to lack of data. We only know that the participants were not hazardous drinkers in the three months prior to the baseline interview. Individuals with history of alcohol abuse or dependence may be at higher risk of relapse when dealing with stressful situations [47]. The risk of relapse among former hazardous drinkers is potentially higher after losing a job during economically challenging times. Predictably, the availability of data on previous risk patterns among participants would have reinforced our results. Finally, Poland was excluded from the analysis because it had a very low incidence of hazardous drinking with respect to other countries and its contextual variables’ values were also quite different. However, a sensitivity analysis including Poland provided similar results at the individual level.

### Changes in alcohol consumption during the late 2000s economic crisis

During the 6-year follow-up period, cumulative incidence of hazardous drinking among middle-aged people was 6.6 per 100 persons, which may correspond to an annual incidence of 1.1 cases per 100 person-years assuming that the incidence was constant over the period of study. This incidence was similar to the one found by Kalousova et al. [19] in a study of 19-64-years-old people from the U.S.: after 2 years of follow-up, 4% of individuals engaged in hazardous drinking (i.e. an annual incidence of 2 cases per 100 persons). Future studies should focus on the risk of initiating a risky pattern of drinking, as it is related to adverse health consequences [48,49]. The evolution towards risky patterns among middle-aged deserve the scaling up of prevention policies specifically in this age group [49]. Our findings show that almost 7% of people became hazardous drinkers during the follow-up period, which is of concern not only because of the harmful health effects, but also because negative health behaviours established or re-established during economic difficulties can persist afterwards and become a life-long habit [19].

We observed that job loss in middle-aged people is a risk factor for hazardous alcohol consumption when comparing to employed people, which is consistent with several studies in active population of all ages [17,18,23]. The analysis including country employment rates and other variables, suggest that this individual factor is not confounded by important variables acting at contextual level. Our finding implies that losing the job may also have an effect on health of the group of economically active people over 50.

Individuals aged 50–65 years face a double vulnerability: social and physical. Firstly, a lower probability of being reemployed than younger ones [30,31] would make individual job loss a relevant factor leading to very important levels of strain or hardships for affected middle-aged individuals during economically challenging times in a context of generalized job insecurity. The diminished expectations of finding a new job would increase their stress, which might raise the risk of increasing the intake of alcohol. Studies on mental health found that job loss is also associated with worsening of mental health in all age groups [4,50]. The stress hypothesis is a likely explanation for our findings [23,51,52], although from our data no causal relationship can be established between being deprived of employment and the increase in alcohol intake. Our finding that the risk of becoming a hazardous drinker increases with age, such that older individuals who become unemployed may be most likely to start to drink hazardously is in line with the decreasing possibilities of finding a job as age grows [30,31]. However, this hypothesis requires validation, and caution is needed when interpreting this finding because our results on the role of age may be sensitive to a differential loss to follow-up.

Secondly, when considering the same intake of alcohol, middle-aged and older individuals are more vulnerable to adverse health effects than younger individuals due to age-related changes [26,27]. Middle-aged people and older adults are more likely than younger groups to suffer health problems that can get worsened by risky drinking patterns, as stroke, high blood pressure, diabetes, or mood disorders [28]. In addition, the increased likelihood of health problems among people over 50, also multiplies the possibility of interaction with medication. Non-fatal injuries have been found to be associated with frequency of drinking and problem drinking in a wide sample of 45–69 year old European men and women [53] and over 2/3 of all alcohol-attributable deaths occurring among the 20–64 year old population of the European Union occur in the 45–64 years olds [49]. There is an increasing public health concern regarding the harmful consequences of alcohol among people from middle age onwards because of the rapid ageing population in many countries worldwide [49,54]. Our finding support the conclusion by Rehm et al. (2010) of having middle-aged as a priority group for intervention and policy [49].

Finally, we observed differences in the cumulative incidence of hazardous drinking among countries. Although these differences were not statistically significant, they varied from 5.1% in Germany to 8.2% in Belgium. The highest absolute incidence observed in Belgium could be related to the fact that prevalence of alcohol consumption in this country, and especially of hazardous drinking, is among the highest of the participating countries, as observed in previous studies [44,55].

Although the economic crisis did not have the worst effects on Belgium, other participating countries which suffered the most severe effects of the economic crisis such as Spain or Italy were among those with the highest incidence of hazardous drinking. In this sense, as we observed in the multilevel analysis, there was an association between the incidence of hazardous drinking and the national unemployment rate and the household disposable income. Both variables were correlated with hazardous drinking (changes in unemployment rate: r = 0.420; increases in household disposable income: r = -0.561). While associations found at contextual level should be interpreted with caution due to the low number of countries in the second level of the multilevel analyses, our results are consistent with previous studies that highlight the relationship between declining macroeconomic conditions and alcohol-related risky behaviours [11]. A possible explanation may be that increased national unemployment rate could generate real or perceived economic insecurity among workers, increasing stress and possibly alcohol consumption [9]. We also found that a subtle increase in household disposable income was associated with higher cumulative incidence of hazardous drinking. This supports the hypothesis that poor macroeconomic conditions might promote risky drinking patterns.

## Conclusions

We have found that shifting from employment to unemployment among people aged 50–65 during economically challenging times was positively associated with the adoption of risky patterns of drinking. Considering that detrimental consequences of alcohol among adults grow with age our finding may have very relevant public health implications in a context of an ageing population pyramid. These findings highlight the need to promote prevention policies and interventions, and to improve access to treatment services during economic recession, especially for the most vulnerable groups such as those facing an unemployment situation in middle-aged people.

## Supporting Information

S1 Table Alcohol advertising restrictions in 2008, according to type of alcoholic beverage and media. Eleven European countries participating in waves 2 and 4 of the Survey of Health, Ageing and Retirement in Europe project (SHARE) conducted in 2006–07 and 2011–12, respectively.
(DOC)

S2 Table Alcohol control policies in 2006, according to country. European countries participating in the waves 2 and 4 of the Survey of Health, Ageing and Retirement in Europe project (SHARE) conducted in 2006–07 and 2011–12, respectively.
(DOC)
