# Supplementary material for: Job Loss, Unemployment and the Incidence of Hazardous Drinking during the Late 2000s Recession in Europe among Adults Aged 50–64 Years
Source: PLoS One. 2015 Oct 7;10(10):e0140017. doi: 10.1371/journal.pone.0140017 (PMC4596847; doi:10.1371/journal.pone.0140017)
Supplement: S1 Table — Eleven European countries participating in waves 2 and 4 of the Survey of Health, Ageing and Retirement in Europe project (SHARE) conducted in 2006–07 and 2011–12, respectively. (DOC) [file pone.0140017.s001.doc]

**Supplementary Table S1.- Alcohol advertising restrictions in 2008, according to type of alcoholic beverage and media. 11 European countries participating in both waves of the Survey of Health, Ageing and Retirement in Europe project (SHARE) conducted in 2006-07 and 2011-12, respectively.**

|  | |  | **Media** | | | | | | | | |  |
| --- | --- | --- | --- | --- | --- | --- | --- | --- | --- | --- | --- | --- |
|  | |  | **National television** | **Cable television** | **National radio** | **Local radio** | **Print media** | **Billboards** | **Cinema** | **Internet** | **Point-of-sale** | **Final score** |
| **Alcohol advertising restrictions** | | |  |  |  |  |  |  |  |  |  |  |
| **Austria** | Restrictions on beer | | 1 | 1 | 1 | 1 | 1 | 1 | 1 | 1 | 1 | **35** |
|  | Restrictions on spirits | | 3 | 3 | 3 | 3 | 1 | 1 | 1 | 1 | 1 |  |
|  | Restrictions on wine | | 1 | 1 | 1 | 1 | 1 | 1 | 1 | 1 | 1 |  |
|  |  | |  |  |  |  |  |  |  |  |  |  |
| **Belgium** | Restrictions on beer | | 1 | 1 | 1 | 1 | 1 | 1 | 1 | 1 | 1 | **27** |
|  | Restrictions on spirits | | 1 | 1 | 1 | 1 | 1 | 1 | 1 | 1 | 1 |  |
|  | Restrictions on wine | | 1 | 1 | 1 | 1 | 1 | 1 | 1 | 1 | 1 |  |
|  |  | |  |  |  |  |  |  |  |  |  |  |
| **Czech Republic** | Restrictions on beer | | 1 | 1 | 1 | 1 | 1 | 1 | 1 | 1 | 1 | **27** |
|  | Restrictions on spirits | | 1 | 1 | 1 | 1 | 1 | 1 | 1 | 1 | 1 |  |
|  | Restrictions on wine | | 1 | 1 | 1 | 1 | 1 | 1 | 1 | 1 | 1 |  |
|  |  | |  |  |  |  |  |  |  |  |  |  |
| **Denmark** | Restrictions on beer | | 3 | 3 | 3 | 3 | 2 | 2 | 2 | 2 | 2 | **66** |
|  | Restrictions on spirits | | 3 | 3 | 3 | 3 | 2 | 2 | 2 | 2 | 2 |  |
|  | Restrictions on wine | | 3 | 3 | 3 | 3 | 2 | 2 | 2 | 2 | 2 |  |
|  |  | |  |  |  |  |  |  |  |  |  |  |
| **France** | Restrictions on beer | | 4 | 4 | 3 | 3 | 3 | 1 | 4 | 4 | 3 | **90** |
|  | Restrictions on spirits | | 4 | 4 | 3 | 3 | 3 | 1 | 4 | 4 | 3 |  |
|  | Restrictions on wine | | 4 | 4 | 4 | 4 | 4 | 1 | 4 | 4 | 3 |  |
|  |  | |  |  |  |  |  |  |  |  |  |  |
| **Germany** | Restrictions on beer | | 3 | 3 | 3 | 3 | 2 | 2 | 3 | 3 | 2 | **72** |
|  | Restrictions on spirits | | 3 | 3 | 3 | 3 | 2 | 2 | 3 | 3 | 2 |  |
|  | Restrictions on wine | | 3 | 3 | 3 | 3 | 2 | 2 | 3 | 3 | 2 |  |
|  |  | |  |  |  |  |  |  |  |  |  |  |
| **Italy** | Restrictions on beer | | 3 | 3 | 3 | 3 | 3 | 3 | 3 | 3 | 3 | **81** |
|  | Restrictions on spirits | | 3 | 3 | 3 | 3 | 3 | 3 | 3 | 3 | 3 |  |
|  | Restrictions on wine | | 3 | 3 | 3 | 3 | 3 | 3 | 3 | 3 | 3 |  |
|  |  | |  |  |  |  |  |  |  |  |  |  |
| **Netherlands** | Restrictions on beer | | 2 | 2 | 2 | 2 | 2 | 2 | 2 | 2 | 2 | **54** |
|  | Restrictions on spirits | | 2 | 2 | 2 | 2 | 2 | 2 | 2 | 2 | 2 |  |
|  | Restrictions on wine | | 2 | 2 | 2 | 2 | 2 | 2 | 2 | 2 | 2 |  |
|  |  | |  |  |  |  |  |  |  |  |  |  |
| **Spain** | Restrictions on beer | | 3 | 3 | 2 | 2 | 2 | 3 | 3 | 3 | 3 | **76** |
|  | Restrictions on spirits | | 4 | 4 | 2 | 2 | 2 | 3 | 3 | 3 | 3 |  |
|  | Restrictions on wine | | 3 | 3 | 3 | 2 | 3 | 3 | 3 | 3 | 3 |  |
|  |  | |  |  |  |  |  |  |  |  |  |  |
| **Sweden** | Restrictions on beer | | 4 | 4 | 4 | 4 | 4 | 4 | 4 | 4 | 3 | **105** |
|  | Restrictions on spirits | | 4 | 4 | 4 | 4 | 4 | 4 | 4 | 4 | 3 |  |
|  | Restrictions on wine | | 4 | 4 | 4 | 4 | 4 | 4 | 4 | 4 | 3 |  |
|  |  | |  |  |  |  |  |  |  |  |  |  |
| **Switzerland** | Restrictions on beer | | 4 | 3 | 4 | 4 | 3 | 3 | 3 | 3 | 1 | **85** |
|  | Restrictions on spirits | | 4 | 4 | 4 | 4 | 3 | 3 | 3 | 3 | 1 |  |
|  | Restrictions on wine | | 4 | 3 | 4 | 4 | 3 | 3 | 3 | 3 | 1 |  |
|  |  | |  |  |  |  |  |  |  |  |  |  |
| Source: Adapted from Bosque-Prous *et al.* [41]. Data are from the Global Information System on Alcohol and Health (WHO). The scores given were: 1=no advertising restrictions; 2=voluntary advertising restriction; 3=partial advertising restrictions; 4=advertising ban. The final score was calculated by adding up the scores of every media and type of alcoholic beverage. | | | | | | | | | | | | |
|  |  |  |  |  |  |  |  |  |  |  |  |  |
