# Supplementary material for: Job Loss, Unemployment and the Incidence of Hazardous Drinking during the Late 2000s Recession in Europe among Adults Aged 50–64 Years
Source: PLoS One. 2015 Oct 7;10(10):e0140017. doi: 10.1371/journal.pone.0140017 (PMC4596847; doi:10.1371/journal.pone.0140017)
Supplement: S2 Table — European countries participating in the waves 2 and 4 of the Survey of Health, Ageing and Retirement in Europe project (SHARE) conducted in 2006–07 and 2011–12, respectively. (DOC) [file pone.0140017.s002.doc]

**Supplementary Table S2.- Alcohol control policies in 2006, according to country. European countries participating in the two waves of the Survey of Health, Ageing and Retirement in Europe project (SHARE) conducted in 2006-07 and 2011-12.**

|  | **Subgroup of alcohol control policies** | | | | | | |
| --- | --- | --- | --- | --- | --- | --- | --- |
|  | **Control of production and wholesale of alcohol** | **Control of distribution of alcohol** | **Personal control (age limits)** | **Social and environmental controls  (BAC limits)** | **Public policy** | **Alcohol taxation** | **TOTAL** |
| ***Maximum score*** | *3* | *12* | *4* | *2* | *1* | *16* | ***38*** |
|  |  |  |  |  |  |  |  |
| **Austria** | 0 | 0 | 0 | 1 | 0 | 4 | ***5*** |
| **Belgium** | 0 | 4 | 2 | 1 | 1 | 6 | ***14*** |
| **Czech Republic** | 0 | 0 | 2 | 0 | 0 | 3 | ***5*** |
| **Denmark** | 0 | 5 | 1 | 1 | 0 | 9 | ***16*** |
| **France** | 0 | 4 | 0 | 1 | 1 | 6 | ***12*** |
| **Germany** | 0 | 0 | 0 | 1 | 0 | 4 | ***5*** |
| **Italy** | 0 | 3 | 0 | 1 | 1 | 4 | ***9*** |
| **Netherlands** | 0 | 4 | 2 | 1 | 1 | 8 | ***16*** |
| **Spain** | 0 | 0 | 0 | 2 | 1 | 2 | ***5*** |
| **Sweden** | 0 | 12 | 3 | 2 | 1 | 16 | ***34*** |
| **Switzerland** | 1 | 3 | 2 | 1 | 0 | 5 | ***12*** |

Source: data came from The Bridging the Gap Project [42].
